# Detection of *Mycoplasma bovirhinis* and bovine coronavirus in an outbreak of bovine respiratory disease in nursing beef calves

**DOI:** 10.3389/frmbi.2022.1051241

**Published:** 2022-12-15

**Authors:** Tara G. McDaneld, Aspen M. Workman, Carol G. Chitko-McKown, Larry A. Kuehn, Aaron Dickey, Gary L. Bennett

**Affiliations:** United States Department of Agriculture (USDA), Agricultural Research Service (ARS), U.S. Meat Animal Research Center, Clay Center, NE, United States

**Keywords:** cattle, 16S, microbiome, viral pathogens, nasal, respiratory disease

## Abstract

**Introduction:**

Respiratory disease incidence is intimately associated with an animal’s commensal bacteria populations (microbiome), as microbes that are involved with morbidity and mortality are commonly found in animals with no sign of disease. In addition, viral pathogens affect the immune system and appear to play an integral role in the overall incidence of bovine respiratory disease (BRD); so, an understanding of the interaction of the bacterial and viral pathogens in the upper respiratory tract (URT) may help us to understand the impact of these pathogens on development of BRD. For this research, the overall goal was to characterize bacterial and viral populations in the URT of nursing beef calves at initial vaccination and at the time of a BRD outbreak.

**Methods:**

Nasal swabs from the URT were collected at initial vaccination (average 45 days of age) and again at the time of the BRD outbreak (average 126 days of age). DNA and RNA were extracted from nasal swabs to evaluate bacterial and viral populations in the URT. Whole blood was also collected at the time of the BRD outbreak for determination of complete blood counts. To evaluate the microbiome, hypervariable regions 1 through 3 along the 16S ribosomal RNA (rRNA) gene were amplified by PCR and sequenced using next-generation sequencing (Illumina MiSeq) for identification of the bacterial taxa present. To evaluate the viral pathogens, multiplex reverse transcription real-time polymerase chain reaction and next-generation sequencing (Illumina NextSeq) was completed.

**Results:**

Overall, evaluation of these samples revealed that at the time of the BRD outbreak, all calves were nasally shedding bovine coronavirus and a large percentage had a coinfection with Mycoplasma sp., with Mycoplasma bovirhinis being the predominant species. Neither bovine coronavirus nor Mycoplasma sp. were present at high abundance at the earlier timepoint of initial vaccination. When comparing bacterial population diversity between the two sampling timepoints, alpha diversity was significantly greater at initial vaccination compared to the BRD outbreak (P-value <0.001). Values of leukocytes at the time of the BRD outbreak were also identified to be significantly different between calves with normal or elevated rectal temperatures (P-value <0.05).

**Discussion:**

Analysis of the respiratory microflora in the URT during initial vaccination and a BRD outbreak will provide insight into the distribution of bacterial and viral populations in nursing beef calves.

## Introduction

Bovine respiratory disease (BRD) complex is a multi-factorial disease, which is the most expensive animal disease afflicting the cattle industry (Griffin et al., 2010; USDA, 2013). The disease is termed a “complex” due to its multi-factorial nature, which can have a variety of pathogens involved, sharing common clinical signs. Infectious causes associated with BRD include viral (bovine herpesvirus-1 (BHV-1), bovine coronavirus (BCV), bovine respiratory syncytial virus (BRSV), bovine viral diarrhea virus (BVDV), parainfluenza-3 virus (PI-3), bovine adenoviruses, bovine rhinitis viruses), bacterial (*Mannheimia haemolytica*, *Pasteurella multocida, Histophilus somni, Mycoplasma bovis*), which can often be present in the bovine respiratory tract in the absence of apparent disease (O'Connor et al., 2013; Bell et al., 2014; Guterbock, 2014; Hay et al., 2014; Klima et al., 2014; Neibergs et al., 2014; Taylor et al., 2015; Tizioto et al., 2015; Johnston et al., 2017). It is increasingly evident that microbial populations (microbiomes) associated with distinct anatomical locations in animals have profound effects on disease incidence (Holman et al., 2015; Taylor et al., 2015; Timsit et al., 2016; Johnston et al., 2017; Holman et al., 2017); therefore, evaluation of the animal’s respiratory microflora in the upper respiratory tract (URT) may help us to understand the impact of bacterial and viral pathogens on the incidence of BRD in cattle.

Bovine respiratory disease commonly occurs in the feedlot when stress levels increase in the animals due to weaning, subsequent transport to the feedlot, and comingling (Muggli-Cockett et al., 1992; Dixit et al., 2001; Snowder et al., 2006). As a result, studies focusing on regions of the nasal cavity have concentrated on sampling timepoints after cattle are weaned and diagnosed with BRD in the feedlot (Holman et al., 2015; Timsit et al., 2016; Holman et al., 2017; Johnston et al., 2017). At the feedlot timepoint, Holman et al. (2017) reported the genera *Streptococcus* and *Acinetobacter* were present at day 2 and *Mycoplasma* was present at day 14 after entry into the feedlot (Holman et al., 2017). In a separate study, Holman et al. (2015) also identified the presence of *Mannheimia haemolytica* and *Pasteurella multocida* in feedlot calves diagnosed with BRD. In addition to the bacterial pathogens present in feedlot, Zhang et al. (2021) reported BCV as the most prevalent virus in cattle upon arrival in the feedlot, and Workman et al. (2017) also reported the presence of BCV in cattle diagnosed with BRD after weaning in the feedlot. While the current literature on BRD focuses on cattle in the feedlot, very little has been reported for BRD outbreaks that occur prior to weaning. Therefore, evaluation of the animal’s resident bacterial populations in the upper nasal cavity during a BRD outbreak pre-weaning could provide insight into the role of microbial diversity and will enable study of the role of microbial variation prior to entry into the feedlot. The objective of this study was to characterize the bacterial and viral populations present in the URT of calves during a natural BRD outbreak that occurred in nursing beef calves and study the variation in these populations at the time of the BRD outbreak and an earlier sampling timepoint prior to the outbreak.

## Materials and methods

### Animal populations

Data were collected in 2019, in a subset (fifty-one calves) of the U.S. Meat Animal Research Center USMARC; Clay Center, Nebraska) Selection for Functional Allele (SFA; Bennett et al., 2018) herd. All calves were raised under similar management, receiving standardized vaccinations and diets. The subset of 51 MARC III (1/4 each of Angus, Hereford, Red Poll, and Pinzgauer; Gregory et al., 1991) calves were comingled from birth until weaning. Samples were collected under an experimental outline approved by the U.S. Meat Animal Research Center Animal Care and Use Committee (IACUC Approval No: 24).

### Nasal swabs, rectal temperatures and blood collection

Various methods can be used to sample different sites of the respiratory tract. These include swabbing of the upper nasal cavity or the deep nasal pharyngeal region and bronchoalveolar lavages. For this population, we have chosen to sample the upper nasal cavity as the time and labor associated with bronchoalveolar lavage was not feasible for calves at this age. Previous research has also reported similarity between sampling sites of the respiratory tract (Doyle et al., 2017; McDaneld et al., 2018). As part of a larger and separate sampling project, nasal swabs were collected for 46 of the 51 calves at the time of initial vaccination when calves were approximately 45 days of age. At this time, they received BOVILIS 20/20 VISION 7 with SPUR (MERCK, Madison, NJ) for aid in prevention of disease caused by *Clostridium Chauvoei*, *Cl. septicum*, *Cl. novyi, Cl. sordellii, Cl. perfringens* Types C and D, and *Moraxella bovis* and PYRAMID 5 + PRESPONSE SQ (Boehringer Ingelheim, Ingelheim, Germany) that contained modified-live viruses for strains of IBR, BVDV types 1 and 2, PI3, and BRSV. All 51 calves were then mass treated in August for an unexpected BRD outbreak approximately 80 days later, following the observation that >20% were displaying signs of BRD, including lethargy, nasal discharge, elevated body temperature, and distressed breathing. Of the 51 calves treated at the time of the outbreak, 22 calves (43.1%) had a temperature greater than 102.8°C (range 103.0-106.3°C; **Supplemental Table 1**). No dams for the calves were treated for BRD at the time of the BRD outbreak or prior to or after the outbreak. Nasal swabs, rectal temperatures, and blood were collected at the time of mass treatment for all 51 calves. The calves that were treated had a mean age of 125.6 days, and treatment occurred approximately 45 days prior to weaning. All samples were collected prior to treatment with Draxxin (Zoetis; Parsipanny, NJ). Nasal swabs at initial vaccination (46 calves in total) and the time of the BRD outbreak (51 calves in total) were collected from the URT using 15.24 cm nasal swabs. For sampling, the nose of the animal was wiped clean with a single-use towel if fecal material was present. Two unguarded 15.24 cm nasal swabs were then gently inserted into each nasal cavity at an approximate depth of at least 14 cm. The nasal swabs were then rotated and removed. After collection of the sample, two swabs, one from each nostril, were placed in buffered peptone water with 12% glycerol for subsequent bacterial pathogen evaluation and two swabs were placed in a minimum essential medium (Gibco; Thermo Fisher Scientific, Inc., Waltham, MA) for subsequent viral pathogen evaluation. All samples were stored at -80°C. Rectal temperatures were taken on all calves at the time of treatment. For this study, animals were classified based on rectal temperature at the time of the BRD outbreak to determine if microflora profiles varied based on rectal temperature at the time of the BRD outbreak. Animals with low rectal temperature (LRT) are defined as calves with an approximately standard normal temperature or lower at the time of the BRD outbreak (100.4-102.9°F), while animals with high rectal temperature (HRT) are defined as calves with a rectal temperature higher than the approximately standard normal temperature at the time of the BRD outbreak (103-106.2°F). A single 10 ml blood sample was taken from all calves at the time of the BRD outbreak. One ml of the blood sample was transferred to a 2 ml screw-top tube preloaded with 4% EDTA for subsequent complete blood count (**CBC**) analysis to evaluate blood components.

### Blood sample analysis for CBC

Whole blood samples with EDTA as an anticoagulant (described above) were transported to the laboratory shortly after collection. Samples were maintained on a rocker platform for at least 15 min to come to room temperature. Complete bloods counts were performed on an Element HT5 veterinary hematology analyzer (Heska Corp., Loveland, CO) after a hematology control was measured (CBC-5DRMR Vet, R&D Systems Inc., Minneapolis, MN).

### Data analysis for CBC

For each blood component, comparison was made between LRT calves versus HRT calves running a t-test in GraphPad Prism Version 9.1.0 (GraphPad Software, San Diego, CA).

### DNA extraction for 16S rRNA sequencing for bacterial pathogen evaluation

Total DNA was extracted from swabs of all 51 calves sampled at the time of the BRD outbreak and 46 of these same calves sampled at initial vaccination using a commercial kit (PowerSoil DNA kit; Qiagen, Germantown, MD) and initial DNA quantity was evaluated with a DNA spectrophotometer (DeNovix DS-11 FX Series; Wilmington, DE). A sample of DNA from 16 random LRT calves (of N = 29 LRT calves sampled) and 16 random HRT calves (of N = 22 HRT calves sampled) was then selected for 16S rRNA sequencing for both the initial vaccination and BRD outbreak timepoint. Amplification of the 16S rRNA gene V1 to V3 hypervariable region was then completed for each DNA sample using standard PCR (AccuPrime, Invitrogen, Carlsbad, CA) and primers with index sequences as previously described that amplify hypervariable region 1 through 3 of the 16S rRNA gene (Myer et al., 2015a). Quality and quantity of the resulting 16S rRNA gene amplification was checked on the Fragment Analyzer (Advanced Analytical, Ankeny, IA). By using indexed primers to amplify the 16S rRNA gene, individual samples were pooled into a single sequencing run for both sampling timepoints (initial vaccination and the BRD outbreak) and then sequenced utilizing the MiSeq Illumina Sequencer (Illumina, San Diego, CA) with a MiSeq Reagent Kit v3 to generate 2×300 paired end reads. Samples that did not pass the initial quality score cutoff of Q20>75% for sequence reads were run in a second sequencing run. Approximately 282,992 reads (range 212,374-364,287) were further evaluated for each sample in the pool.

### Data analysis for 16S rRNA data

**Figure 1:**
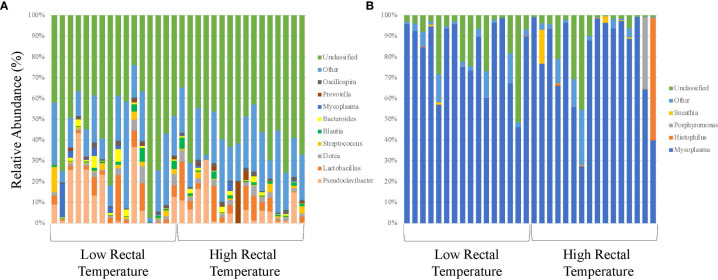
16S rRNA profiles of bacterial genera for calves at initial vaccination and the BRD outbreak. **(A)** 16S rRNA profiles (relative abundance (%)) were evaluated in calves at USMARC at the time of initial vaccination (approximately day 45) in nursing beef calves. Sixteen low rectal temperature (at the time of the BRD outbreak) and 16 high rectal temperature (at the time of the BRD outbreak) calves were selected for 16S rRNA profile analysis. Data present the 9 most relatively abundant genera in the URT microbiota. Any OTU that could not be classified to the genus level are grouped as unclassified and the remaining genera of low abundance are grouped and identified as other. **(B)** 16S rRNA profiles (relative abundance (%)) were evaluated in calves at USMARC at the time of a BRD outbreak in nursing beef calves. Sixteen low rectal temperature (at the time of the BRD outbreak) and 16 high rectal temperature (at the time of the BRD outbreak) calves were selected for 16S rRNA profile analysis. Data present the 4 most relatively abundant genera in the URT microbiota. Any OTU that could not be classified to the genus level are grouped as unclassified and the remaining genera of low abundance are grouped and identified as other.

The paired-end data files for each DNA sample were downloaded from the MiSeq Illumina Sequencer and processed through the MICCA computing environment for sequence data (Albanese et al., 2015). Operational taxonomic units (OTUs) were assembled from high quality sequence reads based on a 97% identity cut off. Taxonomic assignment for the identified OTUs was determined using the Naïve Bayesian assignment based on composition similarity (Garcia-Etxebarria et al., 2014) against the Greengenes database (current version 13_8) and a niche-specific database (Myer et al., 2019). Data were then evaluated for common contaminants (Salter et al., 2014) that may have originated from contaminated reagents or consumables during the DNA extraction. If bacterial genera of common contaminants were identified in the data set, the second swab collected from the animal was extracted for DNA and subsequent 16S rRNA gene amplification. Data reported herein are from taxonomic classifications based on the niche-specific database. Data are presented as a relative abundance (%) of each bacterial genus, phylum, and family in the sample. Any OTU that could not be classified to the taxonomic level are grouped and identified as unclassified and the remaining genera of low abundance are grouped and identified as other. An average of 47.8% and 11% of the OTUs could not be classified to the genus level and are identified as unclassified for the timepoints of initial vaccination and the BRD outbreak, respectively. The OTUs were compared using Phyloseq in R (R Core Team, 2019). The top 9 and 4 bacterial genera in abundance are reported for the initial vaccination and BRD outbreak timepoints, respectively, and remaining genera of low abundance are grouped and identified as other (**Figure 1**). Information of top phylum and family are also reported for the timepoints of initial vaccination and BRD outbreak (**Supplemental Figures 1**, **2**). Alpha diversity metrics (differences within samples; using a Shannon index) were analyzed as pairwise comparison using Wilcoxon rank sum test. Beta diversity (differences among samples) was analyzed using permutational multivariate analysis of variance (PERMANOVA) using the adonis() function and vegan. The DEseq2 package was used to estimate differences in taxa abundance. All these analyses were completed in R.

### qPCR for mycoplasma species

Presence of *Mycoplasma* species *M. bovis, M. dispar* and *M. bovirhinis* was evaluated by qPCR for all 51 calves sampled at the time of the BRD outbreak and 46 of these same calves sampled at initial vaccination. QPCR reactions were performed in duplicate following manufactures instructions for SYBR Green (BioRad, Hercules, CA). Two μl of DNA from previously described DNA extractions was used in each qPCR reaction. Primers and cycling conditions for *M. bovis* were F: 5’-CTT GGA TCA GTG GCT TCA TTA GC-3’, R: 5’-GTC ACT ATG CGG AAT TCT TGG GT-3’, 95°C for 3 minutes, 35 cycles of 95°C for 15 seconds, 55°C for 30 seconds and 72°C for 1 minute. Primers and cycling conditions for *M. dispar* were F: 5’-TTA AAG CTC CAC CAA AAA-3’, R: 5’-GTA TCT AAA GCG GAC TAA-3’, 95°C for 3 minutes, 35 cycles of 95°C for 15 seconds, 54°C for 30 seconds and 72°C for 30 seconds. Primers and cycling conditions for *M. bovirhinis* were F: 5’-GCT GAT AGA GAG GTC TAT CG-3’, R 5’-ATT ACT CGG GCA GTC TCC-3’, 95°C for 3 minutes, 35 cycles of 95°C for 15 seconds, and 60°C for 1 minute. Cycle threshold (**Ct**) values less than 35 were considered positive.

### Data analysis for qPCR of mycoplasma species

Average Ct value and percentage of samples positive for each *Mycoplasma* species was determined for *M. bovis*, *M. dispar* and *M. bovirhinis*. For each *Mycoplasma* species, comparison was made between calves classified as LRT and HRT running a t-test in GraphPad Prism Version 9.1.0. (GraphPad Software, San Diego, CA).

### Detection of viral pathogens by RT-qPCR

Viral RNA and DNA were extracted from nasal swab samples collected from all 51 calves sampled at the time of the BRD outbreak and 46 of these same calves sampled at initial vaccination using a silica-membrane-based nucleic acid purification kit (Qiagen viral mini kit; Qiagen, Germantown, MD). Presence of BCV, BRSV, BVDV, and BHV-1 was evaluated by multiplex reverse transcription real-time polymerase chain reaction (RT-qPCR) using primers, probes, and cycling conditions as previously described (Workman et al., 2017). Cycle threshold (Ct) values less than 38 were considered positive. Positive, negative, and no template controls were included on each run. Comparisons of Ct values between calves classified as LRT and HRT was completed as described above.

### Metagenomic sequencing for viral pathogen detection

Nasal swab medium from samples collected from twenty calves with the highest rectal temperatures at the time of the BRD outbreak were pooled into four groups of five. The average rectal temperature of the calves in the four pools were 105.4°F, 104.2°F, 103.4°F, and 103.1°F, respectively. Pooled nasal swab medium was clarified by centrifugation at 10,000 x g for 2 minutes at 4°C. Viral RNA and DNA were extracted using a silica-membrane-based nucleic acid purification kit as described above (Qiagen viral mini kit; Qiagen, Germantown, MD). Two hundred nanograms of viral nucleic acid was used as input material for the Illumina DNA prep kit to detect DNA viruses. Twenty-five microliters of the eluted viral nucleic acid were treated with Turbo DNAse (Ambion, Austin, TX) according to the manufacturer’s instructions. Two hundred nanograms of the remaining RNA was used as input material for the Illumina TruSeq Stranded RNA sample preparation kit to detect RNA viruses. RNA libraries were constructed as specified by the manufacturer’s protocol without the initial step of poly(A) selection on oligo(dT) beads to allow sequencing of viral genomes lacking poly(A) tails. DNA and RNA libraries were sequenced on an Illumina NextSeq 2000 instrument with a 300-cycle kit to generate 2 x 151-bp paired-end reads and a 200-cycle kit to generate 2 x 101-bp paired-end reads, respectively. An average of 220 million reads per library were sequenced.

### Data analysis for viral sequencing libraries

Adapters were trimmed from raw sequence reads using bbduk (from BBMap version 38.06). Trimmed reads were screened against a host database using HISAT2 (version 2.1.0) and reads not mapping to the bovine reference genome (UMD3.1) were assembled using skesa (version 2.4.0). Assembled contigs were blasted against a local viral database created August 16, 2021, from sequences obtained using NCBI_Mass_Sequence_Downloader (version 4.4) with the advanced query: “Viruses[Organism] NOT wgs[PROP] NOT cellular organisms[ORGN] NOT AC_000001:AC_999999[PACC] AND (“50”[SLEN]: “5000000”[SLEN])”. The taxonomy of the local database sequences was obtained from the NCBI nucl_gb.accession2taxid.gz file and the taxdb database. The local database was deduplicated using seqkit (version 0.10.1) and clumpify (from BBMap version 38.67) and was created and queried using BLAST+ (version 2.10.0). Database queries used the BLAST+ program megablast, an E-value cut-off of 1e-10 and max_target_seqs = 1. BLAST results were then manually screened using a commercial software package (Geneious v2021.2.1; Biomatters, Auckland, New Zealand). Trimmed, host filtered sequence reads or skesa assembled contigs were mapped to the reference viral genomes identified in the BLAST search to determine genome coverage and depth and to rule out spurious mapping which could result in the identification of viruses not truly present in the sample (false positives).

## Results and discussion

A study was completed to identify the respiratory microflora present in the URT and quantify blood components of nursing beef calves during a BRD outbreak at the U.S. Meat Animal Research Center in 2019. Differences among bacterial and viral profiles and blood components were determined by comparing calves with low versus high rectal temps at the time of the BRD outbreak. Bacterial and viral profiles at the time of the BRD outbreak were also compared to profiles at initial vaccination, which was approximately 80 days prior to the BRD outbreak.

### Bacterial pathogen evaluation

**Figure 2:**
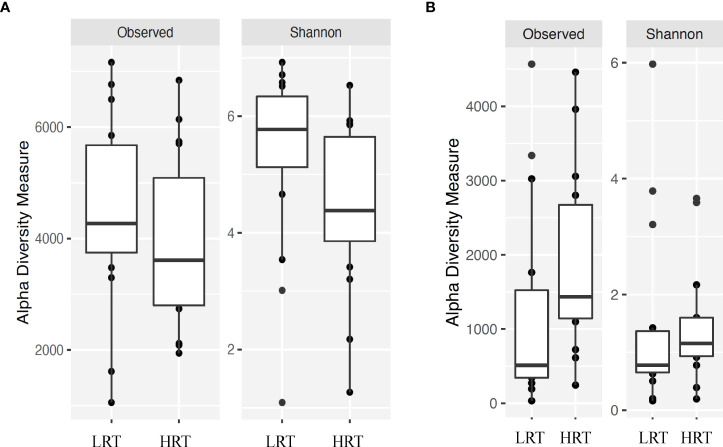
Alpha diversity for bacterial genera for calves at initial vaccination and the BRD outbreak. **(A)** Alpha diversity for bacterial genera at initial vaccination. Low rectal temperature (LRT; at the time of the BRD outbreak) and high rectal temperature (HRT; at the time of the BRD outbreak). **(B)** Alpha diversity for bacterial genera at the time of the BRD outbreak. Low rectal temperature (LRT; at the time of the BRD outbreak) and high rectal temperature (HRT; at the time of the BRD outbreak).

**Figure 3:**
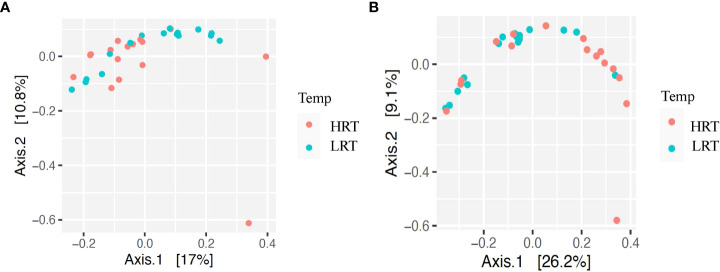
Beta diversity for bacterial genera for calves at initial vaccination and the BRD outbreak. **(A)** Beta diversity for bacterial genera at initial vaccination. Low rectal temperature (LRT; at the time of the BRD outbreak) and high rectal temperature (HRT; at the time of the BRD outbreak). **(B)** Beta diversity for bacterial genera at the BRD outbreak. Low rectal temperature (LRT; at the time of the BRD outbreak) and high rectal temperature (HRT; at the time of the BRD outbreak).

**Figure 4:**
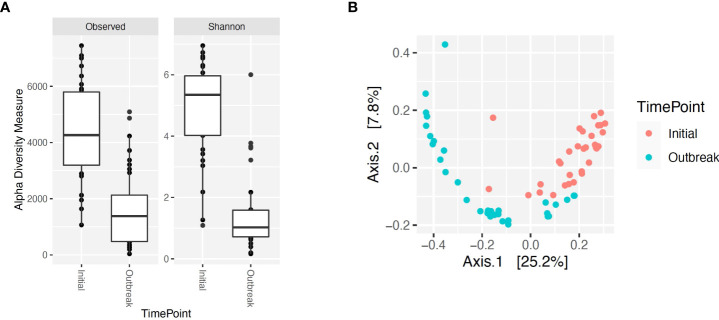
Alpha and beta diversity for bacterial genera for calves at initial vaccination versus the BRD outbreak. **(A)** Alpha diversity for bacterial genera at initial vaccination (Initial) versus the BRD outbreak (Outbreak). **(B)** Beta diversity for bacterial genera at initial vaccination (Initial) versus the BRD outbreak (Outbreak).

Alpha and beta diversity of the bacterial profiles was evaluated to identify bacteria pathogen diversity both within and across nasal samples collected from the URT of calves. When evaluating each timepoint individually, there was no significant difference in alpha diversity (variation within calf samples) at either initial vaccination or the BRD outbreak (**Figures 2A, B**; *P-value* = 0.27 and 0.067, respectively). For beta diversity (diversity across calf samples), while there was no significant difference at initial vaccination (**Figure 3A**; *P-value* = 0.424); however, there was a significant difference in beta diversity at the time of the BRD outbreak (**Figure 3B**; *P-value* = 0.017), indicating variation in the diversity of the bacterial profiles across calf samples at the BRD outbreak timepoint. When specifically comparing alpha and beta diversity between the two time points of initial vaccination and the BRD outbreak, both were significantly different (**Figure 4A**; alpha diversity *P-value* < 0.001; **Figure 4B**; beta diversity *P-value <*0.001) indicating variation in bacterial profiles both within and among samples collected at initial vaccination versus those at the BRD outbreak. This significance in bacterial profile diversity between the two timepoints may be due to the predominance of *Mycoplasma* sp. at the time of the BRD outbreak versus relatively low abundance (<10%) at initial vaccination (**Figure 1**). Additionally, the greater proportion of OTU classified as ‘unclassified’ and ‘other’ for the initial vaccination timepoint may be contributing to the significance in alpha and beta diversity between the two sampling timepoints.

When evaluating the bacterial profiles through 16S rRNA sequencing at the time of the BRD outbreak (expressed as a relative abundance of the bacterial genus in the sample), only OTUs classified as *Firmicutes* at the phyla level and *Lactobacillales* at the class level (these OTUs could not be classified beyond the class level) were significantly different between calves with LRT versus those with HRT (*P*-value = 0.003). For the LRT calves, no OTUs for *Lactobacillales* were identified, however of the 16 HRT calves evaluated, 5 calves had OTUs for *Lactobacillales* ranging from 0.05% to 2.1% of the of the total OTUs present. While the relative abundance of *Lactobacillales* is low in the HRT calves evaluated, previous literature has identified *Lactobacillales* in the microbiome of the URT of turkeys and the lung and in the sputum of children with pneumonia (Pragman et al., 2012; Pettigrew et al., 2016; Kursa et al., 2021). This suggests that *Lactobacillales* may have a role during the BRD outbreak in nursing beef calves, however further research needs to be completed to elucidate this role.

Of the remaining OTUs present for the BRD outbreak timepoint, the most abundant genus in the bacterial profile was *Mycoplasma* sp. (**Figure 1B**). While there was no significant difference in relative abundance of *Mycoplasma* sp. in LRT calves versus HRT calves (*P*-value > 0.05), the average relative abundance was 81% across the samples evaluated (**Figure 1B**) suggesting that *Mycoplasma* sp. is associated with the BRD outbreak. This is further suggested by the data that *Mycoplasma* sp. is at low relative abundance (<10%) at the time of initial vaccination (approximately 45 days of age) when calves were not displaying symptoms of BRD (**Figure 1A**).

With *Mycoplasma* sp. being the most abundant genus in calves evaluated at the time of the BRD outbreak, identification of the specific *Mycoplasma* species associated with the BRD outbreak was determined. Specific *Mycoplasma* species evaluated included *M. bovis*, *M. dispar* and *M. bovirhinis*, which were quantified by qPCR. *Mycoplasma bovis* is traditionally associated with BRD (Fulton, 2009a; Caswell et al., 2010); however, with the advancement of 16S rRNA sequencing, other *Mycoplasma* species including *M. dispar* and *M. bovirhinis* have been identified in the microbiome of cattle with BRD (Timsit et al., 2016; McMullen et al., 2018; McMullen et al., 2020a). For the data presented herein, *M. bovirhinis* was the predominant species present at the time of the BRD outbreak (Ct value < 24.0) with *M. bovis* and *M. dispar* having a lower abundance with a Ct value > 26.0 in both LRT and HRT calves (**Table 1**); however, there was no significant difference in *Mycoplasma* species abundance (based on Ct values) between LRT and HRT calves (*P*-value > 0.05). Percentage of calves positive for *Mycoplasma* sp. was greatest for *M. bovirhinis* at 96.6% (28/29) for LRT and 95.5% (21/22) for HRT calves. *Mycoplasma bovis* and *dispar* were also present in a relatively high percentage of calves at 86.2% (25/29) for LRT and 86.4% (19/22) for HRT calves for *M. bovis* and 89.7% (26/29) for LRT and 90.9% (20/22) for HRT calves for *M. dispar*. In comparison, at the initial vaccination timepoint (approximately 45 days of age), *M. dispar* and *M. bovirhinis* were not detected while *M. bovis* was detected at a low level (average Ct value >30; **Table 1**). Together, these data suggest that *M. bovirhinis* is the predominant *Mycoplasma* species associated with the BRD outbreak in nursing beef calves. While *Mycoplasma* sp. is one of the most common bacterial genera identified in the nasopharynx and lungs of cattle that succumb to BRD (Fulton et al., 2009b; McMullen et al., 2020b), this bacterial genus has also been found commensally in the nasopharynx of apparently healthy, unaffected cattle (Griffin et al., 2010; Zeineldin et al., 2017). Data previously reported from our group shows that *Mycoplasma* sp. is predominant in the URT of calves diagnosed with BRD in the feedlot and is in lower abundance at timepoints prior to weaning in calves that are not displaying signs of BRD (McDaneld et al., 2018; McDaneld et al., 2019). The data presented herein, further support these results as *Mycoplasma* sp. was in relatively low abundance at the initial vaccination timepoint when calves were not displaying symptoms of BRD and then became the predominant genera in calves at the time of the BRD outbreak. There may be mechanisms that trigger commensals such as *Mycoplasma* sp. to convert to pathogenic and virulent bacteria in the nasopharynx and lung and further research needs to be completed to elucidate these mechanisms.

**Table 1:** Average abundance and percent positivity of respiratory pathogens in calves at initial vaccination and mass treatment.

	Initial vaccination				Mass treatment			
	*M. bovis* Average Ct	*M. dispar* Average Ct	*M. bovirhinis* Average Ct	BCVAverage Ct	*M. bovis* Average Ct	*M. dispar* Average Ct	*M. bovirhinis* Average Ct	BCVAverage Ct
LRT	32.6	0.0	0.0	0.0	30.9	26.5	21.0	22.4
HRT	31.3	0.0	0.0	0.0	29.9	27.3	23.6	22.7
	***M. bovis*** **% Positive**	***M. dispar*** **% Positive**	***M. bovirhinis*** **% Positive**	**BCV** **% Positive**	***M. bovis*** **% Positive**	***M. dispar*** **% Positive**	***M. bovirhinis*** **% Positive**	**BCV** **% Positive**
LRT	92.6	0.0	0.0	0.0	86.2	89.7	96.6	100
HRT	89.5	0.0	0.0	0.0	86.4	90.9	95.5	100

Average Ct value and percentage of samples positive for Mycoplasma species M. bovis, M. dispar, M. bovirhinis, and bovine corona virus (BCV) for calves at initial vaccination (46 of 51 calves later treated at mass treatment) and mass treatment (51 of 51 calves treated at mass treatment), relative to rectal temperature status at the time of the mass treatment. Low rectal temperatures at time of the mass treatment (LRT; 100.4-102.9°F) and high rectal temperatures at the time of the mass treatment (HRT; 103-106.2°F).

### Viral pathogen evaluation

RT-qPCR was performed to detect the common respiratory viral pathogens BCV, BHV-1, BRSV, and BVDV in the URT of calves at initial vaccination and again at the time of the BRD outbreak. No viral pathogens were detected at the initial vaccination timepoint. Conversely, BCV was detected in 100% (51/51) of the samples collected during the BRD outbreak; however, there was no significant difference in mean abundance of BCV (based on Ct values) between LRT and HRT calves (*P*-value > 0.05; **Table 1**). A shotgun metagenomics approach was then completed to identify any additional viral pathogens that may be contributing to the disease outbreak. Twenty calves with the highest rectal temperatures were pooled into four groups of five for metagenomic sequencing. An average of 220 million reads were obtained for each library. Reads were trimmed, host filtered, *de novo* assembled, and blasted against a viral database. Bovine coronavirus was the only mammalian virus detected, along with a few viral phage sequences. A complete BCV genome was assembled from each of the four libraries and mapping of the host filtered reads to the reference Mebus BCV genome (GenBank Accession U00735) resulted in a mean genome coverage ranging from 7 to 100x. This suggests that BCV is the main viral pathogen associated with the BRD outbreak in these calves.

In recent years, although still under debate, a primary role of BCV in BRD has been recognized. Bovine coronavirus has been isolated from pneumonic lungs alone or in combination with other respiratory pathogens (Decaro et al., 2008; Saif, 2010), but within the BRD complex this virus is primarily considered an initiator of secondary infections (Ellis, 2019). We previously characterized a pre-weaning BRD outbreak in a similar group of cattle that was associated with the detection of *H. somni* and BCV (Workman et al., 2019). Here, BCV and *M. bovirhinis* were detected in the URT of calves at the time of treatment for BRD. These two studies suggest BCV may be an important contributor to BRD in nursing beef calves.

### Blood component evaluation

Complete blood counts were measured for all calves at the time of the BRD outbreak. Fifty of the calves had total leukocyte (WBC) numbers within normal limits, with one calf from the HRT group having a value below the average range. For the individual types of leukocytes, values for lymphocytes, monocytes, eosinophils, and basophils were within the normal range, as were percent lymphocytes, eosinophils, and basophils (**Supplemental Table 2**). For the remaining blood components, six of the calves had neutrophil numbers lower than the normal range (2 HRT, 4 LRT), 10 calves had percent neutrophils lower than the normal range (4 HRT, 6 LRT), and three calves had percent monocytes higher than the normal range (3 HRT, 0 LRT). The neutrophil levels below the normal range may be due to their migrating from the peripheral blood to the site of the infection (Mortaz et al., 2018). Red blood cell values were higher than the normal range in all calves possibly due to slight dehydration as a result of fever, malaise, and handling (Roland et al., 2014).

When the associations between leukocyte populations and rectal temperature were analyzed (**Supplemental Table 3**), number of monocytes and eosinophils were significantly different between HRT and LRT calves (0.9486 vs 0.7197 and 0.1905 vs 0.1293; *P-value* = 0.018 and 0.005, respectively), with each being greater in HRT calves. The association between percent macrophages and percent eosinophils with rectal temperature was also significant between HRT and LRT calves (9.6% vs 7.5% and 2.0% vs 1.4%; *P-value* = 0.014 and 0.0139, respectively), again with each being greater in HRT calves. While the association between number of lymphocytes and rectal temperature was not significant, the association between percent lymphocytes and rectal temperature was (*P-value* = 0.015), with percentage being greater in the LRT calves.

Lymphocytes are the predominant leukocyte in bovine blood (Roland et al., 2014), therefore an increase in monocyte numbers, or a decrease in lymphocyte numbers would lead to a change in the percentage of both populations of cells. The cytokines responsible for fever and inflammation are produced by lymphocytes and monocytes when they come into contact with lipoproteins within the membranes of *Mycoplasma* (Gondaira et al., 2015; Shimizu, 2016; Wang et al., 2017), thus it is not surprising that an increase in these cell populations during infection would lead to increased temperature/fever, cachexia, and malaise associated with illnesses such as BRD. However, as most of the calves in the present study were nasally shedding BCV and a large percentage were coinfected with *Mycoplasma sp*, it is not possible to determine which pathogen influenced the blood counts.

### Conclusions

Overall, we were able to demonstrate through qPCR and next-generation sequencing that the composition of the core bacterial and viral microflora at the time of the BRD outbreak was dominated by *Mycoplasma* sp. and BCV. While *M. bovirhinis* and BCV were the predominant bacterial and viral agents in all samples evaluated for the data presented herein, recent studies have shown that potential pathogens previously assumed to have only a minor role in BRD pathogenesis have become much more prevalent and influential (Murray et al., 2016a; Murray et al., 2016b; Johnston et al., 2017; Murray et al., 2017), which necessitates further investigation of BRD diagnostics, pathogenesis, and control. Evaluation and reporting of these data of the animal’s resident bacterial and viral populations in the upper nasal cavity from early age through weaning in multiple herds is of importance as it will enable study of the variation of the microbial populations over time resulting in a full picture of the microbiome and how it evolves. Collection and reporting of these data will also improve completeness of bacterial and viral sequence databases that are used for taxa classification. Further, evaluation of the animals’ bacterial and viral populations in the URT in nursing beef calves will improve our understanding of the impact of the microbiome and viral pathogens on incidence of BRD in cattle and may help us to understand incidence of BRD in cattle after weaning in the feedlot.

## Author’s note

Mention of trade names or commercial products in this publication is solely for the purpose of providing specific information and does not imply recommendation or endorsement by the U.S. Department of Agriculture. USDA is an equal opportunity provider and employer.

## Data availability statement

The original contributions presented in the study are publicly available. This data can be found here: https://www.ncbi.nlm.nih.gov/ with accession number: PRJNA908237.

## Ethics statement

The animal study was reviewed and approved by US Meat Animal Research Center Institutional Animal Care and Use Committee.

## Author contributions

Conceptualization of project, TM, AW, and LK; Methodology, TM, AW, and CC-M; Formal Analysis, TM, AW, and AD; Resources, GB, TM, LK; Writing and review, TM, AW, CC-M, LK, AD, GB. All authors contributed to the article and approved the submitted version.

## Supplementary material

Supplementary Figure 1 16S rRNA profiles of bacterial phylum for calves at initial vaccination and the BRD outbreak. S1A. 16S rRNA profiles (relative abundance (%)) were evaluated in calves at USMARC at the time of initial vaccination (approximately day 45) in nursing beef calves. Sixteen low rectal temperature (at the time of the BRD outbreak) and 16 high rectal temperature (at the time of the BRD outbreak) calves were selected for 16S rRNA profile analysis. Data present the 7 most relatively abundant phyla in the URT microbiota. Any OTU that could not be classified to the genus level are grouped as unclassified and the remaining genera of low abundance are grouped and identified as other. S1B. 16S rRNA profiles (relative abundance (%)) were evaluated in calves at USMARC at the time of a BRD outbreak in nursing beef calves. Sixteen low rectal temperature (at the time of the BRD outbreak) and 16 high rectal temperature (at the time of the BRD outbreak) calves were selected for 16S rRNA profile analysis. Data present the 6 most relatively abundant phylum in the URT microbiota. Any OTU that could not be classified to the genus level are grouped as unclassified and the remaining genera of low abundance are grouped and identified as other.

Supplementary Figure 2 16S rRNA profiles of bacterial families for calves at initial vaccination and the BRD outbreak. S2A. 16S rRNA profiles (relative abundance (%)) were evaluated in calves at USMARC at the time of initial vaccination (approximately day 45) in nursing beef calves. Sixteen low rectal temperature (at the time of the BRD outbreak) and 16 high rectal temperature (at the time of the BRD outbreak) calves were selected for 16S rRNA profile analysis. Data present the 8 most relatively abundant families in the URT microbiota. Any OTU that could not be classified to the genus level are grouped as unclassified and the remaining genera of low abundance are grouped and identified as other. S2B. 16S rRNA profiles (relative abundance (%)) were evaluated in calves at USMARC at the time of a BRD outbreak in nursing beef calves. Sixteen low rectal temperature (at the time of the BRD outbreak) and 16 high rectal temperature (at the time of the BRD outbreak) calves were selected for 16S rRNA profile analysis. Data present the 8 most relatively abundant families in the URT microbiota. Any OTU that could not be classified to the genus level are grouped as unclassified and the remaining genera of low abundance are grouped and identified as other.
