# Supplementary figures and images for: Detection of Mycoplasma bovirhinis and bovine coronavirus in an outbreak of bovine respiratory disease in nursing beef calves
Source: Front Microbiomes. 2022 Dec 15;1:1051241. doi: 10.3389/frmbi.2022.1051241 (PMC12993465; doi:10.3389/frmbi.2022.1051241)

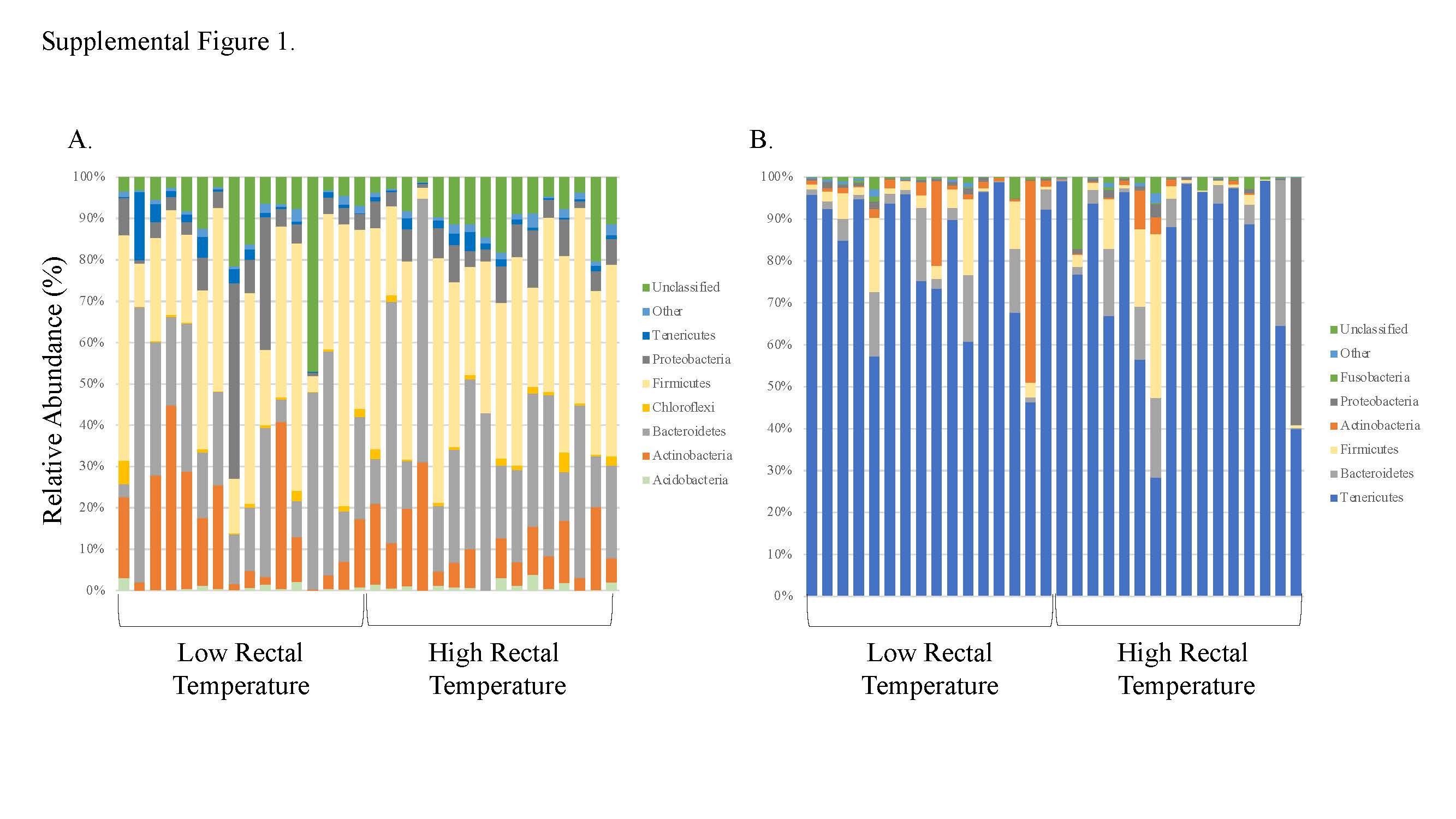

Supplement: Supplementary Figure 1 — 16S rRNA profiles of bacterial phylum for calves at initial vaccination and the BRD outbreak. S1A. 16S rRNA profiles (relative abundance (%)) were evaluated in calves at USMARC at the time of initial vaccination (approximately day 45) in nursing beef calves. Sixteen low rectal temperature (at the time of the BRD outbreak) and 16 high rectal temperature (at the time of the BRD outbreak) calves were selected for 16S rRNA profile analysis. Data present the 7 most relatively abundant phyla in the URT microbiota. Any OTU that could not be classified to the genus level are grouped as unclassified and the remaining genera of low abundance are grouped and identified as other. S1B. 16S rRNA profiles (relative abundance (%)) were evaluated in calves at USMARC at the time of a BRD outbreak in nursing beef calves. Sixteen low rectal temperature (at the time of the BRD outbreak) and 16 high rectal temperature (at the time of the BRD outbreak) calves were selected for 16S rRNA profile analysis. Data present the 6 most relatively abundant phylum in the URT microbiota. Any OTU that could not be classified to the genus level are grouped as unclassified and the remaining genera of low abundance are grouped and identified as other. [file Image_1.jpg]

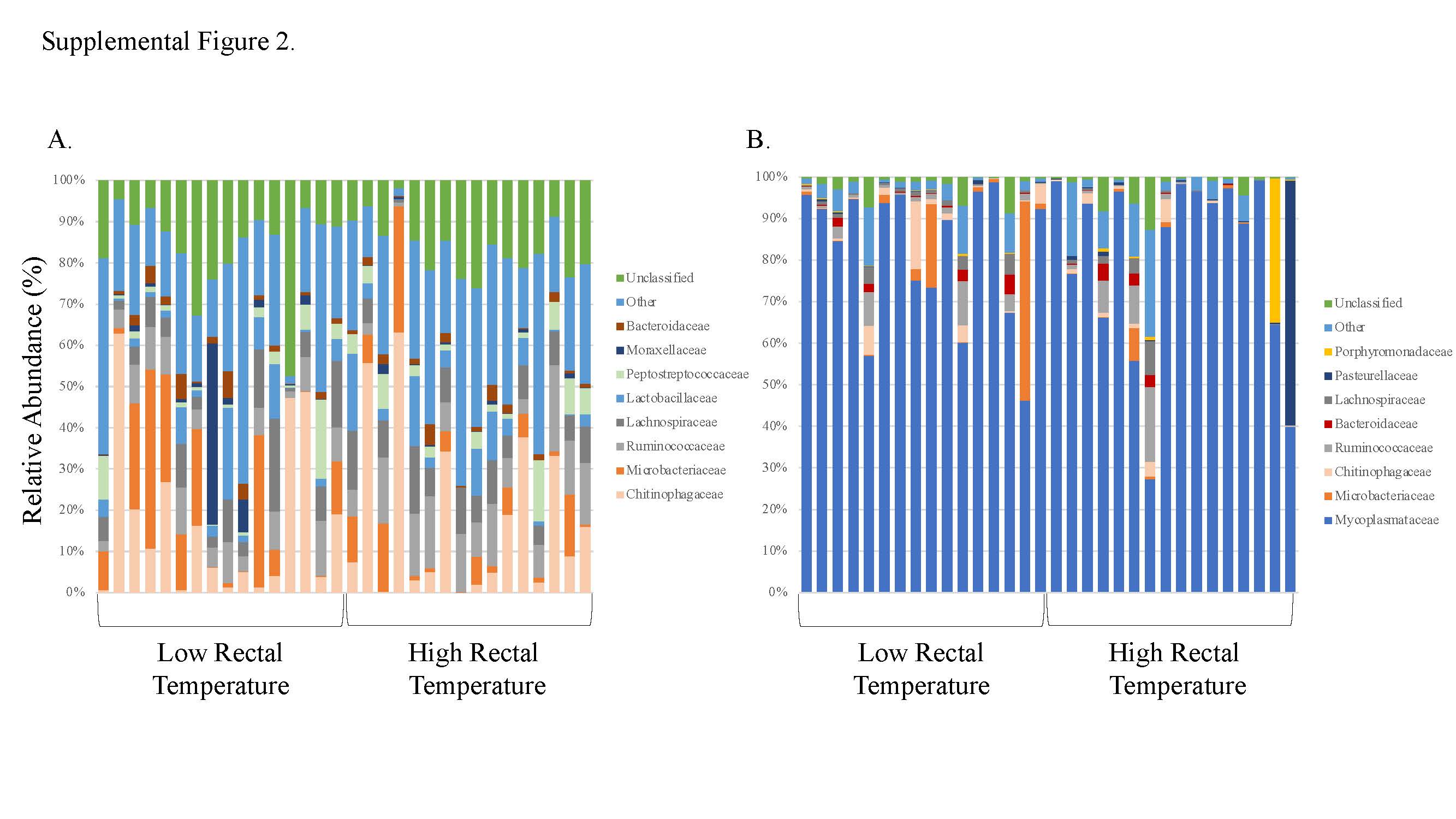

Supplement: Supplementary Figure 2 — 16S rRNA profiles of bacterial families for calves at initial vaccination and the BRD outbreak. S2A. 16S rRNA profiles (relative abundance (%)) were evaluated in calves at USMARC at the time of initial vaccination (approximately day 45) in nursing beef calves. Sixteen low rectal temperature (at the time of the BRD outbreak) and 16 high rectal temperature (at the time of the BRD outbreak) calves were selected for 16S rRNA profile analysis. Data present the 8 most relatively abundant families in the URT microbiota. Any OTU that could not be classified to the genus level are grouped as unclassified and the remaining genera of low abundance are grouped and identified as other. S2B. 16S rRNA profiles (relative abundance (%)) were evaluated in calves at USMARC at the time of a BRD outbreak in nursing beef calves. Sixteen low rectal temperature (at the time of the BRD outbreak) and 16 high rectal temperature (at the time of the BRD outbreak) calves were selected for 16S rRNA profile analysis. Data present the 8 most relatively abundant families in the URT microbiota. Any OTU that could not be classified to the genus level are grouped as unclassified and the remaining genera of low abundance are grouped and identified as other. [file Image_2.jpg]
